# Protocol for Cas9-targeted long-read sequencing in *Globodera pallida* and *Globodera rostochiensis*

**DOI:** 10.1016/j.xpro.2024.103427

**Published:** 2024-11-01

**Authors:** Unnati Sonawala, Lida Derevnina, Sebastian Eves-van den Akker

**Affiliations:** 1 The Crop Science Centre, Department of Plant Sciences, University of Cambridge, CB2 3EA Cambridge, UK

**Keywords:** Bioinformatics, Sequence analysis, Genomics, Plant sciences

## Abstract

We present a protocol to achieve a higher depth of long-read sequencing of region(s) of interest in potato cyst nematodes without amplification using a Cas9-based Nanopore enrichment approach. We describe steps for designing high-fidelity guide RNAs to be used with Cas9 nuclease, extracting high-molecular-weight DNA from the nematodes, and dephosphorylating genomic DNA ends. We then detail procedures for using Cas9-guide RNA complex to make targeted cleavage of the region of interest followed by a Nanopore library preparation.

For complete details on the use and execution of this protocol, please refer to Sonawala et al.^1^

## Highlights

• Protocol for extraction of high-molecular-weight DNA from potato cyst nematode J2s
• Guidance on designing high-fidelity guide RNAs
• Workflow for preparation of Cas9 ribonucleoprotein complexes
• Steps for dephosphorylation of genomic DNA, cleavage, and dA-tailing of target DNA

## Abstract (editor highlights)

Publisher’s note: Undertaking any experimental protocol requires adherence to local institutional guidelines for laboratory safety and ethics.

## Graphical abstract

**Figure:**
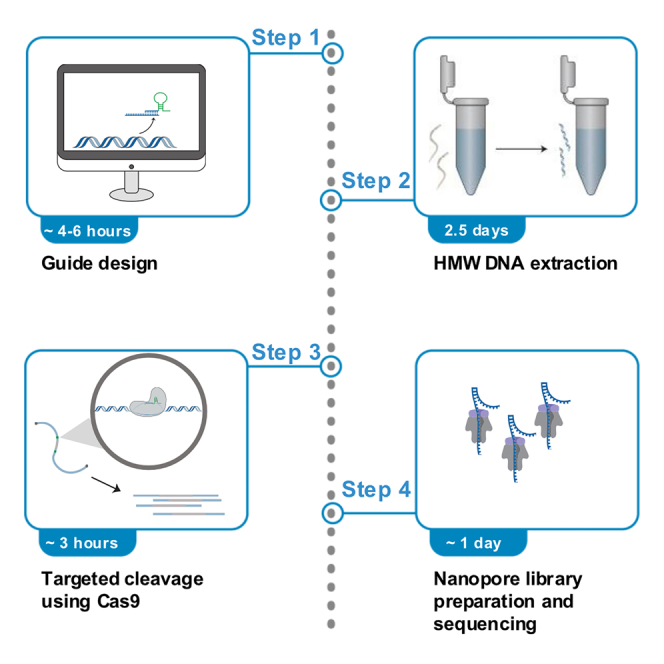

## Before you begin

The following protocol was designed for sequencing HYP1 and HYP3 genes in the potato cyst nematodes, *Globodera pallida* and *Globodera rostochiensis* within a single read using Nanopore sequencing and without using any amplification, but could in principle be applied to other loci. In the case of HYP effector genes, characterizing their genomic variation required higher coverage than that reasonably available via whole-genome sequencing while avoiding polymerase-based amplification steps. This approach uses Cas9-based targeted enrichment to achieve this goal. It can effectively enrich one or more sites in the genome at the same time. This protocol can be applied to enrich any region of interest and potentially be applicable to other nematode species, provided a good-quality reference genome exists for the given organism.

Our protocol starts with designing guide RNAs targeting sites flanking the region of interest and optionally testing their efficacy on PCR-amplified products. High-quality, high molecular weight genomic DNA is dephosphorylated to prevent ligation of adapters. Cas9-guide RNA ribonucleoprotein complex(es) are prepared and used to cleave targeted sites in the freshly dephosphorylated genomic DNA. These cleaved sites are dA-tailed following which a Nanopore ligation-sequencing kit can be used to ligate adapters and load the library on a flow cell for sequencing on a MinIon.

## Key resources table

**Table:** 

REAGENT or RESOURCE	SOURCE	IDENTIFIER
**Chemicals, peptides, and recombinant proteins**		

Alt-R CRISPR-Cas9 tracrRNA	IDT	Cat #1072532
Alt-R CRISPR-Cas9 crRNA, 2 nmol	IDT	N/A
Alt-R S.p. Cas9 nuclease V3	IDT	Cat #1081058
10X rCutSmart buffer	NEB	Cat #B6004S
Shrimp alkaline phosphatase (rSAP)	NEB	Cat #M0371S
Phenol-chloroform-isoamyl alcohol mixture (25:24:1)	Sigma-Aldrich	Cat #77617
Chloroform-isoamyl alcohol (24:1)	Sigma-Aldrich	Cat #C0549
Proteinase K	Promega	Cat # MC5005
AMPure XP beads	Beckman Coulter	Cat #A63882
Taq polymerase	NEB	Cat #M0273S
Quick T4 DNA ligase (available from either NEBNext Companion Module for Oxford Nanopore Technologies or another similar kit)	NEB	E7185

**Critical commercial assays**		

Ligation sequencing kit	Oxford Nanopore Technologies	SQK-LSK112
Qubit dsDNA high sensitivity quantitation kit	Thermo Fisher Scientific	Cat #Q32851

**Deposited data**		

Targeted Nanopore sequencing of HYP loci from *G. pallida* and *G. rostochiensis*	Sonawala et al.^1^	GenBank: PRJNA1078841

**Experimental models: Organisms/strains**		

*Globodera pallida*	James Hutton Institute, UK	Taxon ID: 36090
*Globodera rostochiensis*	James Hutton Institute, UK	Taxon ID: 31243

**Oligonucleotides**		

crRNA for targeted HYP1 and HYP3 Nanopore sequencing	Sonawala et al.^1^	Table S3

**Software and algorithms**		

FlashFry	McKenna and Shendure^2^	https://github.com/mckennalab/FlashFry
Guppy	Oxford Nanopore Technologies	https://community.nanoporetech.com/downloads

**Other**		

Plastic micropestles for 1.5 mL microcentrifuge tubes	Kimble Kontes	Cat # 749521-1590
Nanopore MinION	Oxford Nanopore Technologies	MIN-101b
HulaMixer sample mixer	Thermo Fisher Scientific	Cat #15920D
Nanopore MinION flow cells	Oxford Nanopore Technologies	R10.4, FLO-MIN112

## Materials and equipment

**Table:** Lysis buffer

Reagent	Final concentration	Amount
1 M Tris (pH 8.0)	0.1 M	5 mL
5 M NaCl	0.5 M	5 mL
0.5 M EDTA	50 mM	5 mL
10% SDS	1%	5 mL
ddH_2_O	N/A	30 mL
**Total**	**N/A**	**50 mL**

Store at room temperature. The lysis buffer can be stored for up to a year. Any precipitate formed during storage can be returned to the solution by heating at 37°C–42°C for 15 min and mixing by inversion.

### Elution buffer

10 mM Tris-HCl (pH 8.5). Store at room temperature.

***Alternatives:*** Commercially available elution buffers containing 10 mM Tris (pH 8.0–8.5) with 0.1 mM EDTA can also be used.

## Step-by-step method details

### Design guide RNAs

This step takes you through the process of designing guide RNAs and optionally testing them before Cas9-targeted sequencing.

1. Index your genome to build a database for FlashFry^2^ (https://github.com/mckennalab/FlashFry).

>java -Xmx6000m -jar ∼/FlashFry-assembly-1.10.jar index -tmpLocation ./tmp -reference {genome sequence in fasta format} -database {name of index} -enzyme SPCAS9NGG

2. Discover potential guides in your region of interest.

>java -Xmx6000m -jar ∼/FlashFry-assembly-1.10.jar discover -database {name of index} -fasta {sequence of region of interest in fasta format} -output {output file name}

3. Select a forward guide at the start of the region of interest and a reverse guide at the end of the region of interest. Filter candidates with high-specificity scores and only one perfect match per guide in the genome by filtering for ‘IN_GENOME = 1’.

***Note:*** For this study guides with ‘hsu2013’ scores > 90, off-target matches with at least 3 mismatches using the flag ‘basesDiffToClosestHit’, and those with flags of ‘dangerous_GC’ and ‘dangerous_polyT’ being ‘NONE’ were considered.

4. Order the selected guides as Alt-R CRISPR-Cas9 crRNA (IDT) along with the universal Alt-R CRISPR-Cas9 tracrRNA (IDT).

***Optional:*** It is advisable to select up to three candidates for forward and reverse guides each per region of interest and test them before using a set for sequencing. This can be achieved by designing primers to amplify each of the sites containing candidate forward and reverse guide sites. The amplicon size can vary between 500–1500 bp. The amplicon should preferably flank >200 bp on each side of the guide and the resulting cleaved products should differ by > 100 bp to be able to visualize them efficiently on an agarose gel. To perform the test, the amplicon should be purified using a PCR cleanup column filter kit, followed by the steps for ‘Preparing Ribonucleoprotein Complexes (RNPs)’ and ‘Cleaving’ in this protocol. These Cas9-cleaved PCR products can be run on a 0.1% Agarose gel alongside the original PCR product as a control to test for cleavage efficiency.

### Collection of potato cyst nematode juveniles (J2s) for DNA extraction

5. Collect second stage juvenile plant-parasitic nematodes (J2s) to be used for DNA extraction and wash in 0.01% Tween 20 solution.
6. Centrifuge at >10,000 × *g* and collect up to 50,000 J2s per 1.5 mL microcentrifuge tube.
  a. Remove as much liquid as possible using a P200 and then followed by a P20 pipette.
  b. Flash-freeze in liquid nitrogen and store at −80°C.

***Note:*** It is advisable to scale-up DNA extraction by using multiple tubes containing ∼50,000 J2s instead of increasing the number of J2s in a single tube.

### Total high molecular weight (HMW) DNA extraction from J2s

**Timing: 2.5 days**

This step describes the protocol for extracting HMW DNA from potato cyst nematode J2s. It has been adapted from the following protocol: https://www.pacb.com/wp-content/uploads/2015/09/SharedProtocol-Extracting-DNA-usinig-Phenol-Chloroform.pdf.

7. Homogenize and digest J2s for DNA extraction.
  a. Add 20 μL of lysis buffer to the frozen J2 pellet and grind with a micro-pestle for ∼30 s until the pellet has thawed.
  b. Add 140 μL of lysis buffer and 40 μL of proteinase K. Mix by inversion.
  c. Incubate at 55°C for 18 h, mixing gently but often.
8. Eliminate RNA.
  a. Add 10 μL of RNase A (10 mg/ml, Thermo Fisher Scientific) and incubate at room temperature for 5 min.
  b. Label this as tube 1.
9. First extraction.
  a. Add an equal volume of phenol/chloroform/isoamyl alcohol solution to the lysed J2s (usually 200 μL).
  b. Either vortex tube for 1 min or rotate on a HulaMixer sample mixer for 15 min. The latter is preferred to retain the integrity of HMW DNA.
  c. Spin the tube at >10,000 × *g* for 5 min.
  d. Remove ∼180 μL of the top aqueous solution and place it into a new tube (tube 2).

**CRITICAL:** Avoid touching or disturbing any of the phenol/chloroform/isoamyl alcohol phase.

***Note:*** To retain the integrity of HMW DNA as much as possible, it is important to pipette the aqueous phase gently. It is also preferable to cut the ends of a P1000 pipette tip before using it to pipette the aqueous phase.

10. Second Extraction:
  a. Add 200 μL of the elution buffer to the original tube 1 containing phenol/chloroform/isoamyl alcohol phase.
  b. Either vortex tube for 1 min or rotate on a HulaMixer sample mixer for 15 min.
  c. Spin the tube at >10,000 × *g* for 5 min.
  d. Remove ∼180 μL of the top aqueous solution from tube 1 while avoiding any of the phenol/chloroform/isoamyl alcohol phase. Add it to the previously collected aqueous solution in tube 2.
11. Third extraction/ back extraction:
  a. Add equal volumes of the chloroform/isoamyl alcohol solution (usually ∼360 μL) to tube 2.
  b. Either vortex tube 2 for 1 min or rotate on a HulaMixer sample mixer for 15 min.
  c. Spin tube 2 at high speed for 5 min.
  d. Remove as much of the top aqueous solution as possible while avoiding the chloroform/isoamyl alcohol solution and place it into a new tube, tube 3.
12. Genomic DNA precipitation:
  a. Add NH_4_OAc to tube 3 to a final concentration of 0.75 M. Add 1 μL of glycogen (Thermo Fisher Scientific, 20 mg/ml). Mix the solution well. Add 2.5X volume of 100% ethanol and mix well.
  b. Spin for 20 min in a 4°C centrifuge at top speed. Decant the supernatant carefully without disturbing the pellet.
  c. Wash the pellet by adding 300 μL of 80% ethanol (prepared fresh) and flicking the tube.
  d. Spin for 15 min in a 4°C centrifuge at top speed.
  e. Decant the supernatant and repeat step 12.c-12.d to wash the pellet a second time.
  f. Decant the supernatant and use a P20 pipette to carefully remove excess 80% ethanol. Allow the pellet to air dry for ∼30 min.
  g. Resuspend the pellet in 25–30 μL of elution buffer. Allow the pellet to resuspend overnight at 4°C.
13. Quality and quantity assessment:
  a. Test the purity of the genomic DNA by measuring the ratio of absorbances at 260/280 nm and 260/230 nm using Nanodrop (Thermo Fisher Scientific). The ratios for genomic DNA with high purity should be between 1.8-2.0 and 2.0–2.2, respectively (see troubleshooting problem 2).
  b. Measure the amount using a fluorometric quantification method such as Qubit by following the manufacturer’s protocol (Thermo Fisher Scientific).

### Preparing the Cas9 ribonucleoprotein complexes (RNPs)

**Timing: 45 min**

The following steps for RNP preparation and Cleaving and dA-tailing target DNA have been adapted and modified from the Cas9 targeted sequencing protocol from Nanopore, version: ENR_9084_v109_revR_04Dec2018.

14. Mix all the crRNAs (each at 50 μM) to be used in equal volumes to a total volume of 3 μL.

***Note:*** crRNAs targeting several sites in the genome can be mixed at the same time. Two sites (HYP1 and HYP3 genes in potato cyst nematodes) were targeted with 0.75 μL of forward and reverse crRNAs, each at 50 μM.

15. In a 0.2 mL thin-walled PCR tube mix the following and spin down:

 8 μL duplex buffer.

 2 μL 50 μM tracrRNA.

 2 μL 50 μM crRNA mix.

16. Using a thermal cycler heat the above at 95°C for 5 min and then allow it to cool down to room temperature.
17. In a 1.5 mL microcentrifuge tube mix the following to form Cas9 RNPs.

 10 μL tracrRNA and crRNA mix from step 15.

 10 μL 10X NEB rCutSmart Buffer.

 79.2 μL nuclease-free water.

 0.8 μL HiFi Cas9 (62 μM).

18. Mix by flicking the tube and incubate at room temperature for 30 min. Post-incubation, return the tube to ice until further use.

### Genomic DNA dephosphorylation

**Timing: 1 h 15 min**

19. Mix the following in a 0.2 mL thin-walled PCR tube:

 3 μL 10X NEB rCutSmart Buffer.

 25.5 μL of ∼5–10 μg of HMW genomic DNA extracted from J2s.

20. To the above mixture add 1.5 μL of Shrimp Alkaline Phosphatase (rSAP, 1000 units/ml).
21. In a thermal cycler, incubate at 37°C for 1 h and then at 65°C for 10 min to inactivate the rSAP.

### Cleaving and dA-tailing target DNA

**Timing: 40 min**

22. Prepare a fresh 10 mM dATP solution by mixing 1 μL of 100 mM dATP with 9 μL of nuclease-free water. Mix well by flicking the tube and spin down.
23. To the tube containing 30 μL solution of dephosphorylated genomic DNA add the following:

 10 μL Cas9 RNPs (from step 18).

 1 μL 10 mM dATP.

 1 μL NEB Taq polymerase.

24. In a thermal cycler, incubate the mixture at 37°C for 30 min followed by 72°C for 5 min, and then return to ice.

***Note:*** When performing this step to test the guides using PCR products, the dA-tailing step can be skipped. After incubating the PCR product with Cas9 RNPs at 37°C for 30 min, heat the mixture at 80°C for 5 min to denature the Cas9 enzyme before running on a 0.1% agarose gel.

### Adapter ligation and cleanup & priming and loading of flow cell

25. Follow the protocol for the latest ligation sequencing kit starting at adapter ligation. The protocol may call for a larger volume of DNA than the 42 μL at the end of step 24. Make up to the volume required using nuclease-free water.

***Note:*** SQK-LSK112 kit from Nanopore was used for adapter ligation, AMPure XP bead purification, priming and loading of the flow cell for this protocol. However, this kit has since been discontinued. Since Nanopore kits and protocols are rapidly developed, it is important to follow the steps associated with the latest ligation sequencing kit available.

Furthermore, the efficiency and requirements of future sequencing kits and/or flow cells with new chemistries may vary from those used when developing this protocol. Hence, it is highly useful to optimize the protocol for available sequencing kit using Flongle flow cells if possible. Additionally, this will enable users to gauge enrichment output to library input ratio and hence refine the starting material accordingly.

**CRITICAL:** For the adapter ligation step, most protocols require incubation for 10 min at room temperature. However, we have found that longer incubation times at this step (up to 2 h) increase the number of reads resulting from a library preparation.

## Expected outcomes

The genomic DNA extraction protocol yields ∼3–5 μg of high-molecular-weight DNA from ∼50,000 J2s of potato cyst nematodes. Using ∼5 μg of genomic DNA we achieved a coverage of up to 2500x for the region of interest (HYP1 in *G. pallida* ‘Newton’) compared to ∼200X coverage in whole-genome sequencing.

## Limitations

The success of this protocol depends upon two factors: a) availability (and accuracy) of a genome for the organism of interest to enable the designing of high-fidelity guide RNAs and b) high-quality and high-molecular-weight genomic DNA from the organism. While the genomic DNA extraction protocol described here is a good starting step for many nematode species, some may need optimization, especially at the lysis step (See troubleshooting problem 1 for issues with low DNA quantity). Furthermore, *in silico* metrics of guide RNA efficacy may not always reflect actual results. This can be improved by optionally testing multiple guide RNAs on PCR-amplified products prior to using them for library preparation (See troubleshooting problem 3).

## Troubleshooting

### Problem 1

Low quantity of genomic DNA.

### Potential solution

This could either be a result of incomplete lysis of nematodes or insufficient amount of starting material. Ensure the J2s are broken down well with a micropestle and the tissue is completely digested at the end of incubation with lysis buffer and proteinase K. One microliter of the lysing solution can be placed on a Petri dish and viewed under a dissecting microscope the next morning to test for complete digestion. A well-lysed sample should have little to no whole/ broken worms.

### Problem 2

Low quality of genomic DNA.

### Potential solution

This is often due to phenol carryover. Care should be taken when pipetting the aqueous phase. Oftentimes, if enough starting material is available, it is better to leave behind some aqueous phase than to get too close to the interphase.

### Problem 3

Low enrichment of region of target gene(s) or region of interest.

### Potential solution

This is most likely due to the low-efficacy of guide RNAs. Follow the optional step of testing multiple guides under Design guide RNAs and use guides with high cleavage efficacy. Despite good efficacy when testing with PCR products, guide RNAs may vary in their efficacy when used with whole genomic DNA. Use another set of guide RNAs if necessary.

### Problem 4

Little to no cleavage when testing guides with PCR products.

### Potential solution

This is potentially due to variation in guide RNA efficacy but could also be due to errors in the genome used to design guide RNAs. Ensure a high-quality genome assembly is used and test multiple guide RNAs.

### Problem 5

Low throughput from Nanopore sequencing.

### Potential solution

Targeted sequencing will result in lower throughput in general than whole genome sequencing. In general, we recommend starting with at least ∼5 μg of HMW DNA and ensuring guide efficacy see problem 3. Furthermore, in addition to scaling up starting material, two or more libraries can be prepared, the flow cell can be washed using a flow cell wash kit, and a second library can be loaded to increase total throughput.

## Resource availability

### Lead contact

Sebastian Eves-van den Akker (se389@cam.ac.uk).

### Technical contact

Unnati Sonawala (us275@cam.ac.uk).

### Materials availability

This study did not generate new unique reagents.

### Data and code availability

• The genome for G. rostochiensis ‘Ro1’ and raw reads for targeted sequencing of HYPs from both, G. rostochiensis ‘Ro1’ and G. pallida ‘Newton’, are available under GenBank: PRJNA1078841.
• The genome for G. pallida ‘Newton’ is available under GenBank: PRJNA702104.

## Acknowledgments

Work on plant-parasitic nematodes at the University of Cambridge is supported by DEFRA license 125034/359149/3 and funded by 10.13039/501100000268BBSRC grants BB/R011311/1, BB/S006397/1, and BB/X006352/1; a Leverhulme grant RPG-2023-001; and a UKRI Frontier Research grant EP/X024008/1.

## Author contributions

U.S. performed the experiments and data analysis; U.S. and S.E.-v.d.A. drafted the manuscript. All authors read and approved the final manuscript.

## Declaration of interests

The authors declare no competing interests.
